# Preeclampsia Onset, Days to Delivery, and Autism Spectrum Disorders in Offspring: Clinical Birth Cohort Study

**DOI:** 10.2196/47396

**Published:** 2024-04-17

**Authors:** Sarah Carter, Jane C Lin, Ting Chow, Mayra P Martinez, Chunyuan Qiu, R Klara Feldman, Rob McConnell, Anny H Xiang

**Affiliations:** 1 Department of Research and Evaluation Kaiser Permanente Southern California Pasadena, CA United States; 2 Department of Anesthesiology and Perioperative Medicine Baldwin Park Medical Center Kaiser Permanente Southern California Baldwin Park, CA United States; 3 Department of Obstetrics and Gynecology Baldwin Park Medical Center Kaiser Permanente Southern California Baldwin Park, CA United States; 4 Department of Preventive Medicine Keck School of Medicine University of Southern California Los Angeles, CA United States

**Keywords:** autism spectrum disorders, autism, clinical management, diagnosis, expectant management, fetal exposure, fetal, management, preeclampsia, pregnancy, pregnant women, risk

## Abstract

**Background:**

Maternal preeclampsia is associated with a risk of autism spectrum disorders (ASD) in offspring. However, it is unknown whether the increased ASD risk associated with preeclampsia is due to preeclampsia onset or clinical management of preeclampsia after onset, as clinical expectant management of preeclampsia allows pregnant women with this complication to remain pregnant for potentially weeks depending on the onset and severity. Identifying the risk associated with preeclampsia onset and exposure provides evidence to support the care of high-risk pregnancies and reduce adverse effects on offspring.

**Objective:**

This study aimed to fill the knowledge gap by assessing the ASD risk in children associated with the gestational age of preeclampsia onset and the number of days from preeclampsia onset to delivery.

**Methods:**

This retrospective population-based clinical cohort study included 364,588 mother-child pairs of singleton births between 2001 and 2014 in a large integrated health care system in Southern California. Maternal social demographic and pregnancy health data, as well as ASD diagnosis in children by the age of 5 years, were extracted from electronic medical records. Cox regression models were used to assess hazard ratios (HRs) of ASD risk in children associated with gestational age of the first occurrence of preeclampsia and the number of days from first occurrence to delivery.

**Results:**

Preeclampsia occurred in 16,205 (4.4%) out of 364,588 pregnancies; among the 16,205 pregnancies, 2727 (16.8%) first occurred at <34 weeks gestation, 4466 (27.6%) first occurred between 34 and 37 weeks, and 9012 (55.6%) first occurred at ≥37 weeks. Median days from preeclampsia onset to delivery were 4 (IQR 2,16) days, 1 (IQR 1,3) day, and 1 (IQR 0,1) day for those first occurring at <34, 34-37, and ≥37 weeks, respectively. Early preeclampsia onset was associated with greater ASD risk (*P*=.003); HRs were 1.62 (95% CI 1.33-1.98), 1.43 (95% CI 1.20-1.69), and 1.23 (95% CI 1.08-1.41), respectively, for onset at <34, 34-37, and ≥37 weeks, relative to the unexposed group. Within the preeclampsia group, the number of days from preeclampsia onset to delivery was not associated with ASD risk in children; the HR was 0.995 (95% CI 0.986-1.004) after adjusting for gestational age of preeclampsia onset.

**Conclusions:**

Preeclampsia during pregnancy was associated with ASD risk in children, and the risk was greater with earlier onset. However, the number of days from first preeclampsia onset to delivery was not associated with ASD risk in children. Our study suggests that ASD risk in children associated with preeclampsia is not increased by expectant management of preeclampsia in standard clinical practice. Our results emphasize the need to identify effective approaches to preventing the onset of preeclampsia, especially during early pregnancy. Further research is needed to confirm if this finding applies across different populations and clinical settings.

## Introduction

Autism spectrum disorder (ASD) is a neurodevelopmental condition characterized by repetitive behaviors and difficulties in communication, social behavior, and sensory processing [1]. ASD is typically diagnosed in early childhood [2], with prevalence rising [3]. In 2020, it was estimated that 2.8% of children in the United States had an ASD diagnosis at 8 years old, an increase from 0.7% in 2000 and 2.3% in 2018, with rates varying by sex, race, and ethnicity [3]. ASD is characterized by a range of symptoms, behaviors, and severity; children with autistic disorders, Asperger syndrome, or pervasive developmental disorder not otherwise specified can be diagnosed with ASD [4]. The etiology of ASD is multifactorial, and genetic factors only explain a small proportion [5]. Early life exposures, including adverse maternal health conditions during pregnancy, such as obesity and diabetes, and environmental pollutants, such as near-roadway air pollution and particulate matter 2.5, are all shown to be associated with the development of ASD [5-9].

One common pregnancy complication is preeclampsia, a hypertensive condition defined by placental vascular deterioration and maternal liver or kidney dysfunction [10]. As preeclampsia is an inflammatory condition complicating 2% to 8% of pregnancies worldwide each year [11], it is important to understand its effect on offspring’s development and long-term health. Previous studies have reported associations between maternal hypertensive disorders and an increased risk of ASD in children [12-15]. Hypertensive disorders of pregnancy, which include preeclampsia, were shown to be associated with adverse childhood neurodevelopment [16,17] and a 2-fold increase in ASD risk in childhood [15]. A meta-analysis reported a higher risk of ASD in the offspring of mothers with preeclampsia than those with gestational hypertension [18]. Previous studies also reported that preeclampsia is associated with an increased risk of ASD [6,19], as well as with preterm birth, which is a risk factor for ASD [20]. However, it is unknown whether the increased ASD risk associated with preeclampsia is due to preeclampsia onset or clinical management of preeclampsia after the onset. Identifying the risk associated with preeclampsia onset and exposure is important to provide evidence supporting the care of high-risk pregnancies and to reduce adverse effects on offspring.

Evidence suggests that preeclampsia results from impaired vascularization of the placenta, with inadequate diastolic uterine arteries causing insufficient blood flow to the placenta and increased systemic maternal inflammation [21]. The primary treatment for preeclampsia is delivering the baby or managing the condition until the best time to deliver the baby [22-24]. Depending on the severity, health care providers will closely monitor symptoms, including blood pressure, platelet count, and fetal growth, as well as renal and hepatic function, and hypertensive medications can be added to control blood pressure [24]. Current preeclampsia treatment guidelines advise expectant management for preeclampsia symptoms until 34 or 37 weeks gestation [24], depending on the severity of symptoms, such that preeclampsia occurring early could be expected to continue for several weeks before delivery.

The purpose of this study is to fill the knowledge gap by examining whether the number of days from preeclampsia onset to delivery in clinical standard practice is associated with an increased risk of ASD in offspring, considering the gestational age of preeclampsia onset. This study will provide important information concerning clinical guidelines for the management of preeclampsia and the effort to minimize the impact on offspring. Data are derived from a large, representative clinical birth cohort with comprehensive electronic medical records (EMR).

## Methods

### Study Population

This population-based retrospective birth cohort study used mother-child pairs of singleton births at Kaiser Permanente Southern California (KPSC) hospitals between January 1, 2001, and December 31, 2014. Of 414,463 mother-child pairs, 49,875 were excluded for lack of Kaiser Permanente membership by the age of 1 year or death of the child by the age of 1 year. The final cohort for this study was 364,588 mother-child pairs. Children were followed through KPSC EMR from birth until age 5 years. The KPSC health care system includes a diverse population of 4.5 million members throughout Southern California, and member demographic data reflect that of local census tracts [25]. Maternal social demographic and pregnancy health data, as well as the child’s ASD diagnosis, were extracted from KPSC’s integrated EMR.

### Outcome: ASD Diagnosis in Children at or Before the Age of 5 Years

The outcome of this study was whether a child had an ASD diagnosis before or at the age of 5 years and the age of the initial diagnosis in KPSC EMR. We chose the follow-up of children up to the age of 5 years because the majority of ASD cases were diagnosed by the age of 5 years, and prenatal exposure is likely to manifest its adverse effect on early developmental disorders. The diagnosis of ASD is recorded in EMR using the *International Classification of Diseases* (*ICD*) codes developed by the World Health Organization. KPSC transitioned from *ICD-9* codes *to ICD-10* on October 1, 2015. In EMR records dated before October 1, 2015, ASD diagnoses were identified by *ICD-9* codes 299.0, 299.1, 299.8, and 299.9. After that date, ASD diagnosis was identified by *ICD-10* codes F84.0, F84.3, F84.5, F84.8, and F84.9. The diagnostic codes included autistic disorders, Asperger syndrome, and pervasive developmental disorder not otherwise specified but excluded Rett syndrome or childhood disintegrative disorder. ASD diagnosis was determined if codes were present in the EMR at 2 or more separate health visits. This method was previously validated by an expert chart review with a positive predictive value of 88% [8,26-28].

### Exposure: Maternal Preeclampsia During Pregnancy

Maternal preeclampsia during pregnancy was identified by *ICD-9* codes 642.50-642.54, 642.60-642.64, and 642.40-642.44 and included eclampsia and hemolysis, elevated liver enzymes, low platelet count (HELLP) syndrome, a serious form of preeclampsia characterized by a low platelet count and elevated liver enzymes [29]. Preeclampsia is diagnosed when blood pressure measurements are >140 systolic or 90 diastolic and when a urine test reports >0.3 g of protein in 24 hours [24,30]. Severe cases involve blood pressure >160 systolic or 110 diastolic and 1 or more of the following: persistent headache, vision changes, thrombocytopenia (platelet count <100,000/mcL), impaired liver function, progressive renal insufficiency (serum creatinine >1.1 mg/dL or doubling of serum creatinine not explained by other known renal disease), and pulmonary edema [24,30]. The date of first occurrence (ie, onset defined for this study) of preeclampsia during pregnancy was extracted, and the corresponding gestational week of preeclampsia onset was calculated by subtracting the date of last menstrual period (LMP) from the date of first occurrence recorded in the EMR. Preeclampsia onset was categorized into the following three groups: (1) <34 weeks (diagnosis between 24 weeks 0 days and 33 weeks 6 days); (2) 34-37 weeks (34 weeks 0 days to 36 weeks 6 days); and (3) ≥37 weeks (≥37 weeks 0 days). This categorization reflected known gestational ages for fetal viability at birth and clinical management guidance for preeclampsia. The gestation of 24 weeks has been reported to be the lower boundary for infant survival [31,32], and expectant management guidelines aim to maintain pregnancies until 34 or 37 weeks, depending on the timing of preeclampsia onset and the severity of symptoms [30]. Expectant management duration was calculated as the number of days between the date of preeclampsia onset and the date of delivery.

### Covariates

Covariates selected to adjust for potential confounding were maternal age, self-reported race, ethnicity, and education; prepregnancy obesity, diabetes, and smoking during pregnancy; history of comorbidity (≥1 diagnosis of heart, lung, kidney, liver disease, or cancer); offspring sex; and census tract–level household income at child’s first birthday. These covariates were potential risk factors associated with child’s ASD risk, as shown in previous studies [21,24]. The birth year was also included as a covariate to account for trends of increasing ASD prevalence over the study period [8]. Maternal obesity was defined as a prepregnancy BMI ≥30 kg/m^2^. Maternal prepregnancy BMI was calculated using maternal height and weight recorded in EMR from the date closest to LMP, with a window of 6 months before and 3 months after LMP [33]. Diabetes during pregnancy included preexisting type 1 or type 2 diabetes and gestational diabetes mellitus diagnosed before 26 weeks, as these were previously associated with ASD risk in this study sample [26,27].

### Statistical Analyses

Outcome variables were child’s ASD diagnosis by the age of 5 years and the age of ASD diagnosis. Exposure variables of interest were the gestational age of preeclampsia onset and days from preeclampsia onset to delivery. The gestational age of preeclampsia onset was analyzed as a continuous variable as well as a categorical variable categorized as <34 weeks, 34-37 weeks, and ≥37 weeks. Duration from preeclampsia onset to delivery was analyzed both as a continuous variable and a categorical variable, using the median number of days between onset and delivery for each onset group as the cutoff. Maternal and child characteristics by preeclampsia exposure status were reported as median and IQR for continuous variables and total number (n) and proportion (%) for categorical variables. Wilcoxon rank-sum tests and chi-square tests were used to assess differences in maternal and child characteristics between preeclampsia exposures.

Associations between preeclampsia exposure and child’s ASD risk were assessed using Cox regression models. Robust standard errors were used to correct for potential correlation between siblings born to the same mothers. Associations were quantified as hazard ratios (HRs) with 95% CI. We first assessed the risk of ASD associated with preeclampsia exposure (yes vs no), followed by assessing ASD risk associated with gestational age of onset within the preeclampsia group and comparing the risk of ASD in the <34 weeks, 34-37 weeks, and ≥37 weeks onset groups relative to the unexposed. We then examined ASD risk associated with duration from onset to delivery among those with preeclampsia exposure, adjusting for gestational weeks of the first occurrence of preeclampsia. All models adjusted for birth year, maternal age, self-reported race and ethnicity, educational qualifications, prepregnancy obesity, diabetes, smoking during pregnancy, census tract–level household income at child’s first birthday, and child’s sex. Birth year was modeled as a penalized spline to account for the nonlinear relationship between birth year and outcomes. We also assessed the roles of gestational age at delivery and birth weight as pathways to risk associated with early onset of preeclampsia and duration from onset to delivery by further adjusting these 2 variables.

Statistical significance was set at *P*<.05. All statistical analyses were performed in R software (version 3.6; R Foundation for Statistical Computing).

### Ethical Considerations

This study was approved by KPSC Institutional Review Boards (review #12075), with individual participant consent waived. All data analyzed were deidentified. No compensation was offered to individual participants. There was no community involvement in the study.

## Results

Table 1 presents cohort characteristics by maternal preeclampsia status. Of the 364,588 children included in this study, 16,205 (4.4%) were exposed to preeclampsia in utero (Table 1). The preeclampsia group had more nulliparous women than the non-preeclampsia group (8238/16,205, 50.8% vs 124,405/348,383, 35.7%). Maternal age at delivery, race, ethnicity, and educational qualifications, as well as census-tract household income and smoking behavior during pregnancy, did not differ between the 2 groups. Larger proportions of women with preeclampsia had diabetes preexisting or diagnosed at ≤26 weeks gestation (2194/16,204, 13.5% vs 20,147/348,383, 5.8%), obesity (4242/16,205, 26% vs 53,210/348,383, 15%), and histories of comorbidities (2694/16,205, 16.6% vs 45,291/348,383, 13%) than women who did not have preeclampsia. Sex was comparable among children of mothers with and without preeclampsia. Both median gestational age at delivery (38 weeks vs 39 weeks) and median birth weight (3005 g vs 3390 g) were lower in children of mothers with preeclampsia than in children of mothers without preeclampsia.

**Table 1:** Cohort characteristics by preeclampsia exposure. Tests for differences in each characteristic between the 2 groups were statistically significant at *P*<.001.

Characteristics				No preeclampsia (n=348,383)		Preeclampsia (n=16,205)
**Maternal characteristics**						
	Age at delivery (years), median (IQR)			30.1 (26.0-34.1)		30.3 (25.6-34.8)
	**Parity, n (%)**					
		0	124,405 (35.7)		8238 (50.8)	
		1	113,130 (32.5)		3602 (22.2)	
		>2	90,515 (26)		3069 (18.9)	
		Unknown	20,333 (5.8)		1296 (8)	
	**Race and ethnicity, n (%)**					
		White	88,014 (25.3)		3591 (22.2)	
		Hispanic	177,877 (51.1)		8491 (52.4)	
		Other	82,492 (23.7)		4123 (25.4)	
	**Education, n (%)**					
		High school or unknown	129,909 (37.3)		6057 (37.4)	
		Some college	101,839 (29.2)		5240 (32.3)	
		College and postgraduate study	116,635 (33.5)		4908 (30.3)	
	Census-tract household annual income (US $), median (IQR)			55,700 (41,500-74,000)		53,500 (39,800-70,300)
	Diabetes^a^, n (%)			20,147 (5.8)		2194 (13.5)
	Obesity^b^ (BMI ≥30 kg/m^2^), n (%)			53,210 (15)		4242 (26)
	History of comorbidity^c^, n (%)			45,291 (13)		2694 (16.6)
**Child characteristics**						
	Male, n (%)			178,388 (51.2)		8510 (52.5)
	Gestational age (weeks), median (IQR)			39 (38-40)		38 (36-39)
	Birth weight (g), median (IQR)			3390 (3080-3710)		3005 (2450-3462)

^a^Maternal diabetes includes preexisting type 1 diabetes (T1D) and type 2 diabetes (T1D) and gestational diabetes mellitus (GDM) diagnosed at ≤26 weeks (no preeclampsia—T1D: 574, 0.2%; T2D: 9150, 2.6%; GDM ≤26 weeks: 10,423, 3%; preeclampsia—T1D: 137, 0.8%; T2D: 1128, 7%; GDM ≤26 weeks: 929, 5.7%)

^b^Height and weight at each clinical visit were not recorded in Kaiser Permanente Southern California electronic medical records until late 2006; therefore, maternal obesity data were missing for children born between 2001 and 2006. Maternal prepregnancy BMI was categorized as obese (BMI ≥30 kg/m^2^), nonobese (BMI <30 kg/m^2^), and unknown (including mothers with unavailable BMI information). Maternal obesity proportion excludes women with missing BMI data (no preeclampsia: missing BMI sample size=135,139; preeclampsia=6229)

^c^Maternal comorbidity was defined as ≥1 diagnosis of heart, lung, kidney, liver disease, or cancer.

Of the 16,205 pregnancies with preeclampsia, 2727 (16.8%) first occurred at <34 weeks, 4466 (27.6%) first occurred between 34 and 37 weeks, and 9012 (55.6%) first occurred at ≥37 weeks. When assessing days from first occurrence to delivery, expectedly, mothers with onset at <34 weeks had the greatest number of days from first occurrence to delivery, with 31.6% (863/2727) having ≥10 days between onset to delivery in this group. For first onset at ≥37 weeks, 78.8% (7104/9012) delivered in ≤1 day. Median days from preeclampsia onset to delivery were 4 (IQR 2,16) days, 1 (IQR 1,3) day, and 1 (IQR 0,1) day for onset at <34, 34-37, and ≥37 weeks, respectively.

A total of 7194 (2.1%) of the 348,383 children were diagnosed with ASD by the age of 5 years: 2.9% (465/16,205) in the preeclampsia exposed and 1.9% (6729/348,383) in the unexposed groups, with an HR of 1.36 (95% CI 1.23-1.49) of ASD risk for exposed versus unexposed after adjusting for birth year, maternal age, race, ethnicity, education, history of comorbidity, prepregnancy obesity, diabetes, smoking during pregnancy, offspring sex, and census-tract household income (Table 2). When considering preeclampsia exposure by gestational week of first onset, earlier preeclampsia onset was associated with greater ASD risk (*P*=.003 for testing ASD association with gestational age of preeclampsia onset among the preeclampsia group). The HRs were 1.62 (95% CI 1.33-1.98), 1.43 (95% CI 1.20-1.69), and 1.23 (95% CI 1.08-1.41) for onset at <34, 34-37 weeks, and ≥37 weeks, respectively, relative to the unexposed group (Table 2).

**Table 2:** Hazard ratios (95% CI) of child’s ASD risk associated with preeclampsia exposure and diagnosis at different gestational weeks, relative to unexposed. Adjusted for birth year, maternal age, race, ethnicity, education, prepregnancy obesity, diabetes, smoking during pregnancy, offspring sex, and census-tract household income (per US $10,000).

Variable		Hazard ratio (95% CI)	*P* value
Preeclampsia		1.36 (1.23-1.49)	<.001
**Gestational age at diagnosis**			
	<34 weeks	1.62 (1.33-1.98)	<.001
	34-37 weeks	1.43 (1.20-1.69)	<.001
	≥37 weeks	1.23 (1.08-1.41)	.002

Among those exposed to preeclampsia, the number of days from first onset to delivery was not associated with ASD risk. When analyzed as a continuous variable adjusting for preeclampsia onset, the HR of ASD risk associated with days from onset to delivery was 0.995 (95% CI 0.986-1.004; Table 3). When analyzed as a categorical variable cut at median days stratified by gestational week of preeclampsia onset, the HR associated with days above the median relative to at or below the median days for each onset group were 1.16 (95% CI 0.74-1.82) for onset at <34 weeks; 1.11 (95% CI 0.74-1.65) for onset between 34 and 37 weeks; and 1.23 (95% CI 0.85-1.78) for onset at ≥37 weeks (Table 3).

**Table 3:** Hazard ratios (HRs; 95% CI) of child’s ASD risk associated with the number of days from preeclampsia diagnosis to delivery among those exposed to preeclampsia. Adjusted for birth year, maternal age, race, ethnicity, education, comorbidities, prepregnancy obesity, diabetes, smoking during pregnancy, offspring sex, and census-tract household income (per US $10,000).

Variable		HR (95% CI)	*P* value
Time from diagnosis to delivery as a continuous variable, in days^a^		0.995 (0.986-1.004)	.28
**Time from diagnosis to delivery as a categorical variable stratified by gestational week of preeclampsia diagnosis^b^**			
	<34 weeks	1.05 (0.70-1.57)	.81
	34-37 weeks	1.08 (0.77-1.51)	.65
	≥37 weeks	1.26 (0.93-1.71)	.13

^a^Adjusted for gestational age at preeclampsia diagnosis in weeks, as a continuous variable.

^b^The hazard ratio represents the risk associated with days from diagnosis to delivery above the median relative to equal or below the median distribution within each group (<34 weeks: median 4, IQR 2.16 days); (34-37 weeks: median 1, IQR 1.3 day); (≥37 weeks: median 1, IQR 0.1 day).

Early preeclampsia diagnosis was positively correlated with early gestational age at delivery (*r*=0.90) and lower birth weight (*r*=0.70), but the number of days from onset to delivery was not correlated with gestational age at delivery (*r*=–0.01) or birth weight (*r*=–0.005). Further adjusting for gestational age at delivery and birth weight reduced HRs to 1.19 (95% CI 0.97-1.48) for onset at <34 weeks and 1.27 (1.07-1.50) for onset between 34 and 37 weeks but did not change the HR for preeclampsia onset ≥37 weeks (1.24, 95% CI 1.08-1.41). The number of days from onset to delivery remained unassociated with ASD risk in offspring after adjustment for gestational age at delivery and birth weight.

## Discussion

### Overview

In this large sample and multiethnic clinical cohort study, children exposed to preeclampsia in utero were at higher risk of ASD than children who were not exposed, with a greater risk for children exposed to preeclampsia earlier in pregnancy. However, among children exposed to preeclampsia, there were no significant associations of risk of ASD with the number of days from first onset to delivery. This was analyzed both as a continuous variable adjusting for gestational age of preeclampsia first onset and as a categorical variable cut at the median number of days and stratified by gestational age of preeclampsia first occurrence. These results suggest that a child’s risk of ASD associated with preeclampsia is not increased by expectant management of preeclampsia in standard clinical practice.

To our knowledge, this study is the first to assess whether the reported ASD risk associated with maternal preeclampsia was due to preeclampsia onset or clinical management of preeclampsia during pregnancy. Current clinical management of preeclampsia advises maintenance of pregnancies until 34 or 37 weeks gestation, depending on the week of diagnosis and preeclampsia severity [24]. Expectant management of preeclampsia requires balancing maternal medical needs with intrauterine developmental requirements and reduction of infant comorbidities associated with preterm birth [34,35]. Planned preterm delivery of pregnancies complicated by preeclampsia improves maternal outcomes but increases the risk of neonatal admissions for prematurity [36]. Expectant management of moderate preeclampsia before 37 weeks gestation has been reported to extend pregnancies without increasing neonatal comorbidities; however, studies have reported the risk of stillbirth [37] and neonatal mortality [38] associated with expectant management for severe preeclampsia diagnosed before 34 weeks gestation. The results presented in our study demonstrate that early onset of preeclampsia is associated with a greater risk of ASD in offspring; however, clinical management for preeclampsia does not add additional risk, potentially eliminating one comorbidity of concern associated with preeclampsia exposure and expectant management.

Prenatal preeclampsia may affect child neurodevelopment by altering the maternal environment during fetal maturation. Preeclampsia has been observed to influence maternal immune activation [39], increasing circulation of maternal proinflammatory cytokines, and contributing to higher levels of oxidative stress, which have been associated with divergent neurodevelopment in children [40]. Studies have reported differences in the etiology of early and late-onset preeclampsia [41,42], as well as varying risks of adverse outcomes associated with the timing of exposure [43,44]. Early-onset preeclampsia may be driven by placental dysfunction [45], which occurs when a placental abnormality restricts blood flow, potentially due to suppression of estrogen-related receptor-gamma leading to vascular abnormalities [46]. Late-onset preeclampsia could be primarily a maternal hypertensive condition [45]. Placental inflammation and vascular dysfunction have been linked to an increased risk of ASD in children [47,48]. Therefore, differences in ASD risk by gestational week of onset may be explained by the influence of preeclampsia on specific stages of fetal neurodevelopment [19].

In our study, earlier onset of preeclampsia had larger HRs than onset in later pregnancy, which is consistent with results from a study of ASD risk and preeclampsia, which reported an increased risk of ASD in children exposed to preeclampsia earlier in pregnancy [19]. Adjustment for gestational age at delivery and birth weight attenuated the risk of ASD after early preeclampsia in our study, suggesting that there may be different mechanisms underlying the risk of ASD by the timing of preeclampsia exposure. Exposure to preeclampsia has been reported to be associated with an increased risk of ASD in offspring [49]. Preeclampsia is also associated with preterm birth, another risk factor for ASD [20]. Our results are consistent with previous results that prematurity and low birth weight are risk factors for ASD [6,20] and extend them to show that early preeclampsia is one of the root causes for prematurity, low birth weight, and associated ASD risk.

Our results concerning the increased risk of ASD associated with maternal preeclampsia during pregnancy are consistent with previous findings. However, the novel finding in our study is that the number of days between preeclampsia’s first occurrence and delivery was not associated with ASD risk in offspring after taking the gestational age of preeclampsia onset into account. Thus, the results of our study suggest that clinical expectant management of preeclampsia after diagnosis does not increase the risk of ASD in offspring. As this is the first study to report these results, future research is needed to determine whether this finding is consistent in other populations and to assess whether these associations vary by type of preeclampsia treatment or severity of illness. Our results provide evidence demonstrating the need to identify effective approaches to prevent the onset of preeclampsia, especially during early pregnancy, to mitigate risk not only to mothers but also to offspring.

### Strengths and Limitations

A strength of this study is its large, clinical, and longitudinal birth cohort with characteristics reflecting census tract–level social and demographic information of Southern California. Comprehensive EMR data with detailed pregnancy history and dates allowed us to assess preeclampsia by gestational week of diagnosis and duration from diagnosis to delivery, and to adjust for relevant covariates. The continuity of care at KPSC minimizes the risk of ascertainment bias in exposures and outcomes. We think the assessment of expectant management of preeclampsia on ASD risk is novel.

This observational study has some limitations. The results presented here do not establish a causal link between preeclampsia exposure and ASD risk. The severity of preeclampsia was not explicitly considered due to the lack of a clear definition of severity in EMR. However, diagnosis to delivery periods allowed some inference about severity, as severe cases were likely to be delivered more quickly than moderate cases [24]. Genetic information was unavailable; therefore, we were unable to control for genetic contributions to ASD risk. There may be other perinatal risk factors or postnatal environmental exposures not adjusted for in these analyses.

### Conclusion

In this population-based retrospective clinical birth cohort study, exposure to preeclampsia in utero was associated with an increased risk of ASD in offspring, with a greater risk for children of mothers with preeclampsia occurring earlier during pregnancy. However, among children of mothers with preeclampsia, the number of days between preeclampsia diagnosis and delivery was not associated with increased ASD risk. Our study suggests that clinical management of pregnancies with preeclampsia does not increase the risk of ASD in offspring. Future research into the prevention of preeclampsia is still needed.

## Abbreviations

ASD: autism spectrum disorder
EMR: electronic medical record
HELLP: hemolysis, elevated liver enzymes, low platelet count
HR: hazard ratio
ICD: International Classification of Diseases
KPSC: Kaiser Permanente Southern California
LMP: last menstrual period

## Data Availability

The data set analyzed in this study is not publicly available as it was drawn from electronic medical records.
